# Building an innovative Chagas disease program for primary care units, in an urban non- endemic city

**DOI:** 10.1186/s12889-019-7248-5

**Published:** 2019-07-08

**Authors:** Ana Cristina Pereiro, Silvia Gold

**Affiliations:** Fundación Mundo Sano, Paraguay 1535, 1061 Buenos Aires, Argentina

**Keywords:** Chagas disease, Primary healthcare, Access barriers, Implementation process, Public-private intervention, Neglected disease

## Abstract

**Background:**

On an absolute basis, Argentina is the country with the largest affected population with Chagas Disease (ChD). This constitutes a significant public health issue. As a consequence of Argentina’s migratory patterns, there has been a significant increase of affected population in urban centers. An innovative project for early diagnosis and timely treatment of ChD was designed for Municipal Primary Care Facilities of La Plata City, a non- endemic area, in line with a proposal from the Pan-American Health Organization. The project was a public –private intervention. The objectives of this study were to demonstrate the feasibility of the primary healthcare level for early diagnosis and timely treatment of ChD; to design and implement a tailor made program and to innovate in a public-private association.

**Methods:**

The healthcare barriers for early diagnosis and timely treatment for the population with ChD of La Plata were analyzed. The four dimensions described by Peters et al. (Ann N Y Acad Sci 1136:161–71, 2008) were used. The baseline was measured during a previous pilot project and the same items were evaluated at the end of 2017. The model from Damschroder et al. (Implement Sci 4:50, 2009) was used during the implementation process.

**Results:**

With all the information gathered during this investigation, a “patient-centered” model was designed. During the program, 17,894 people were serologically tested for ChD, 1,394 were positive and 1,035 were treated. Additionally, 3,750 children from 46 public schools were evaluated for risk factors of ChD.

**Conclusions:**

This project showed the feasibility of the primary healthcare level for early diagnosis and timely treatment of ChD. Tailor made programs and public-private associations should be considered for vulnerable populations in emerging economies in order to enhance efforts and obtain better results. This program may be replicated in other countries of Latin America were Chagas is a main public health issue and, with the corresponding adaptations, for other neglected diseases as well.

**Electronic supplementary material:**

The online version of this article (10.1186/s12889-019-7248-5) contains supplementary material, which is available to authorized users.

## Background

On an absolute basis, Argentina is the country with the largest affected population with Chagas Disease (ChD) [1]. This constitutes a significant public health issue for three distinct reasons: 1) ChD’s high morbidity and mortality rate, 2) the fact that the affected population typically belongs to low income segments that often lack access to timely medical diagnosis and treatment, and 3) the high economic and social costs generated by the disease [2–4]. The reasons that explain why Argentina is the country with the largest affected population are various and multi-factorial. Among these many factors, vector surveillance and control as well as structural poverty, are of particular relevance in understanding why ChD is so prominent in Argentina.

Neither vector surveillance and control policies, nor developmental programs have been successful enough in achieving their respective goals in Argentina. Public health policies have been highly erratic in nature, therefore vector surveillance and control strategies were not always consistently implemented to be effective. A study by experts in the field claims that “between 1962 and 1990, 100% coverage was achieved with insecticides, while only 20% of the epidemiological surveillance activities were consistently conducted. The National Chagas Program started with 2000 technicians in 1964, but subsequently dropped to roughly 600 technicians in 1990. The annual household treatment rate dropped from 216,000 in 1978 to just 37,000 in 1985, before recovering to nearly 200,000 in 1993” [5–7]. Developmental policies were weak and intermittent as shown by the analysis of national statistics where the percentage of poverty grew from 4.6% in 1974 [8] up to 32.2% in 2016 [9]. As a consequence, up to now, Argentina has not been able to qualify as a country free of the vector nor has reduced the ChD burden of disease.

ChD has historically been associated with rural populations. However, as a consequence of migratory patterns in place in Argentina since the 1940s (migration from rural areas to larger cities), there has been a significant increase of affected population in urban centers. On the other hand, Argentina also received a large migration from bordering endemic countries such as Bolivia and Paraguay. Currently, the city of Buenos Aires (with no vector) is the city with the largest infected population [10].

For decades, ChD has been associated with rural areas and this is one of the reasons why early diagnosis was not usually performed in big city hospitals. [11, 12]. Poor diagnostic and treatment rates could also be due to the presence of therapeutic controversies among experts related to the effectiveness of etiological treatments during the chronic stage of the disease [13]. Additionally, policy makers were not reactive towards this changing environment and did not focus on the proliferation of specialized centers to address the increased theoretical demand for ChD treatment. For example, by 2009, there were only two specialized Centers in ChD in Buenos Aires: the National Institute of Parasitology, Dr. Mario Fatala Chaben, and the Center of Parasitology from the R. Gutierrez Hospital. All the patients were encouraged to go to those centers. In a country with such a vast territory and demographic dispersion, the lack of specific service providers constitutes an important access barrier to health care provision. Moreover, historically, off the shelf programs introduced by international funding organizations were implemented in heterogeneous regions of Argentina. These were clearly insufficient and unadaptable to the different local scenarios and therefore did not contribute positively to the problem.

The Argentine healthcare system is complex, overlapped, and highly fragmented [14]. Argentina has a wide offer of Public Hospitals with Governmental Management (PHGM) that are free of charge and were originally developed to provide universal coverage. The initial investment in medical infrastructure made by Ramon Carrillo, the first Minister of Health [15] in the 1950s, was record setting and doubled the installed capacity of medical facilities in the country. When R. Carrillo left the ministry, new authorities began a process of financial decentralization of PHGMs, from the national government to provincial governments. However, follow-up investments necessary to maintain the quality standards of PHGMs were seldom made, mainly in those provinces with scarce public budgets, coincidentally, those where ChD is endemic. The labor union health security system borne during the 50’s chose private hospitals for their members as well as private insurance companies. With the rise of both entities, PHGMs were set aside for people with low income [16].

From 1940 onwards and driven in the 80’s by the Alma Ata Declaration, primary care units grew throughout the country. By 2002, nearly 10,000 such centers were distributed across the 24 Argentinean provinces. Although having a diverse structure in terms of technical and human resources, they constituted an important healthcare provider for patients affected with chronic diseases or minor and frequent acute illnesses.

During 2010, the Pan American Health Organization (PAHO) requested (CD50/16 y CD50.R17) that its constituent nations provide medical access to the ChD affected population through Primary Healthcare Centers. Concurrently, Mundo Sano Foundation (MS), a non-governmental organization focused on the prevention of Neglected Tropical Diseases (NTD), began promoting early diagnosis and timely treatment through Municipal Primary Care Facilities (MPCF) in La Plata City, capital of the Province of Buenos Aires, a non-endemic area. One of the main goals of the project was to reduce or eliminate the healthcare barriers for diagnosis and treatment of ChD and to demonstrate the feasibility of primary care as an adequate level for this purpose. A pilot project was implemented in 2010 [17] in order to explore the healthcare barriers and any potential threat to the design or implementation of the final program.

This article describes the analysis of the healthcare barriers for early diagnosis and timely treatment of ChD in the MPCFs of La Plata. It also explores the implementation process of the city wide roll out to 46 MPCFs after the pilot project ended. Both aspects permitted the consolidation of a public policy providing ChD diagnosis and treatment to thousands of people with high quality medical care.

The importance of this study lies in being the first tailor made program for the primary healthcare level implemented by a public-private association in order to favor the access to medical care for people with Chagas Disease.

## Methods

A Framework Convention between MS and the Municipality of La Plata (MLP) was signed during 2010. Initially, a pilot project was designed and implemented in three MPCFs and three rural schools during 2011–2013 [17]. The three MPCFs and the three rural schools were chosen by the local authorities. They were located in an area far away from the city, where many migrants were living. All the population attending the MPCFs for any reason was asked to answer a few questions related with risk factors for ChD (i.e those who came from an endemic area, had received blood transfusions or were born from an infected mother). Pregnant women were systematically tested for ChD. In case of positive results, all their relatives were tested. The diagnostic and therapeutic processes were in line with those recommended by the National Guidelines for ChD. A short survey was designed for children attending public schools in order to determine the existence of risk factors for ChD (Additional file 1). Those children considered at risk were suggested to seek a pediatrician or a general practitioner at the MACFs. Many data for the construction of the baseline and for the final project design were gathered during the pilot project.

The pilot project’s results (Table 1) encouraged local authorities to roll out MS’s ChD diagnosis and treatment program to the 46 MPCFs in La Plata City and so a new Framework Convention between both organizations was signed in order to develop the extended program. The development of the extended program was based on the analysis of the healthcare barriers for early diagnosis and timely treatment of the population with ChD and prior to implementation the model for Consolidated Framework for Implementation Research (CFIR) was applied. The healthcare barriers for early diagnosis and timely treatment of the population with ChD of La Plata were analyzed by performing several visits and interviews to members of the health team and to patients seen at each MPCF in order to evaluate healthcare barriers from both sides: demand and supply. The four dimensions described by Peters, D et al. [18] – geographic accessibility, acceptability, availability and financial accessibility – were used and some criteria were adapted to ChD specificities in order to make it more appropriate for the analysis of this project (Table 2). All the criteria shown in Table 2 were listed in a questionnaire. The aim was to explore their existence or lack thereof and the level of implementation (i.e. existence of guidelines but not implemented, policy and macro environment, geographic accessibility, availability and financial accessibility). All the questions were made by the same researcher and double checked with local authorities, health teams and in some aspects with patients from the MPCFs. The aspect acceptability was not explored. This model was selected because of its feasibility for low and middle- income countries and the experience from other countries (18). The baseline was measured during the pilot project and the same items were evaluated at the end of 2017.

For the implementation process, the CFIR model from Damschroder LJ et al. [19] was used. This model is composed of five major domains: intervention, characteristics, outer setting, inner setting, characteristics of the individuals involved and the process of implementation. This framework was selected because of its feasibility to be used in complex and multi-level structures. The assessment was performed during the pilot project and during the startup of the project. All the criteria shown in Table 3 were listed in a questionnaire. Questions were made by one researcher to local authorities, local health teams and head directors of local hospitals. The aim was to explore the general context and the intervention characteristics. Minor changes were applied (i.e. some few items were grouped for their evaluation) in order to make it more suitable for the analysis and scope of this project.

Based on the analysis performed above, a patient centered model was designed for diagnosis and treatment with the premise “the healthcare needs to move, but not the patient”. The municipality made two vehicles available for this purpose, and two technicians for obtaining blood samples. MS trained and paid for two electrocardiograph technicians. For better planning, MS purchased two ECGs with telemedicine and PDF format studies that could be sent by e-mail to local networks. Additionally, MS hired a cardiologist to evaluate and report all the ECGs carried out across the 46 MPCFs. ECG results were printed and carried to each MPCF so patients could receive them in a timely fashion.

Patients were evaluated on a weekly basis during the first month of treatment and on a bi-weekly basis during the second month of treatment. The purpose of the high frequency with which patients were evaluated during the first 2 months of treatment was to ensure that the medication was administered correctly and to minimize the occurrence of adverse reactions. Social workers conducted home visits to patients who failed to attend check-ups in order to confirm that treatment was being adequately carried out. Those excluded from the antitrypanosomal drugs treatment were either pregnant, over 50 years old or had other sever pathologies (cancer, liver or kidney failure, etc.).

MS recruited physicians working for the La Plata Municipality by paying them an extra incentive for performing extraordinary tasks such as oversight of the new organizational model and supervision of the diagnosis and therapeutic process. All the human resources working in the MPCFs were permanent and hired by La Plata Municipality. Finally, the design included a MS senior physician to assist health teams, if necessary and to solve any problem or doubt. A 24-h communication line was available for the entire network of MPCFs.

As in the pilot study, schoolchildren were also included and the municipality rolled out the program to all the public schools of La Plata. Therefore, all children were given the ChD survey and those that presented risk factors were tested for infection in the MPCFs. Those that tested positive were treated. Ultimately, a subset of patients that were also serological evaluated consisted of those children with risk factors, women who attended primary healthcare centers to conduct routine checkups for ongoing pregnancies, relative from patients who had previously tested positive for ChD, and patients who suffered from diverse clinical problems while presenting epidemiological risks (i.e. those who came from an endemic area, had received a blood transfusion or we born from an infected mother).

## Results

The population from the pilot project had a high prevalence of ChD. This is probably because they were migrants from high endemic areas. All patients were treated since they fulfilled requirements of the National ChD guidelines and there were no drop-outs.

During the extended project (2014–2015) the number of people tested and treated increased. In 2016, and coinciding with elections and the local authority’s rotation, treatment rates fell but then increased during 2017 (Table 1).

**Table 1:** People tested, positive and treated

Year	People tested	Sero +	Treated	Without treatment for medical reasons^b^	Without treatment
2011^c^	112	40	40	0	0
2012^c^	135	50	50	0	0
2013^c^	228	91	91	0	0
2014	5902	439	414	23	2
2015	5545	381	354	21	6
2016^a^	2787	216	37	41	138
2017	3175	177	49	91	37
Total	17894	1394	1035	176	183

^a^Local authorities’ rotation

^b^ Pregnancy, > 50 years, cancer, other severe illnesses, etc.

^c^Pilot project period

Given that MS’s project goal was to reduce or eliminate access barriers to healthcare for vulnerable populations, a specific evaluation to detect access barriers was carried out, before the project was implemented across the 46 MPCFs to which the program was rolled out in La Plata City. For better understanding, the baseline prior to project implementation in 2010 was added as well as the achievements, if any, accomplished by the end of October 2017 (Table 2).

**Table 2:** Access barriers related to Policy and Macro-Environment and Geographic accessibility. Baseline (2010) and results 7 years after implementation (2017)

Dimensions	Criteria	Baseline(2010)	2017
Policy and Macro-Environment			
Legislation	• Health guaranteed by the Constitution • Existence of specific law for ChD • Other supportive laws	• Although constitutionally guaranteed, partially implemented • Law for ChD voted in 2007, partially implemented • Without monitoring actions	• Although constitutionally guaranteed, partially implemented • Implemented as a Municipal Program • Permanent monitoring process
Health Management	• National guidelines for ChD diagnosis and treatment	• Published but not implemented	• Implemented as a core part in the Program • A guide for patients with ChD attending to the MPCF was elaborated
	• Existence of a national and/or provincial Network of health services	• None	• A local network with the hospitals from La Plata was created-although informal
Social involvement in ChD	• Presence in the local media • Presence in local activities • Visited schools • Surveys in schools (children 6 and 12 years)	• Absent and unknown • Absent • None • None	• Activities in all the public schools of La Plata • Participation in local fairs with stands and basic information • 46 • 3750 children
Geographic Accessibility			
User’s location	• Roads	• Few good roads, many households with difficult access during rainy days	• Health assistants prepared to go to distant places if necessary
	• Communication and public transport	• Scarce public transport to hospitals	• Patient centered model, bringing services to MPCF with no need to travel
Service location	• Nearness to patient’s households	• Near but with inconvenient opening hours and long waiting times	• Some MPCF could change opening hours
Availability			
Health workers Training and outcomes	• Trained in diagnosis and treatment • Patients tested • Patients positive • Number of patients treated • Number and specialties	• No experience in treatment • None • None • None • Enough general practitioners. Only 2 cardiologists	• Highly qualified in diagnosis and treatment • 17894 • 1394 • 1035 • Enough general practitioners with central service of cardiology operated by tele-medicine
Drugs Equipment	• Drug stocks • Needed for biochemical diagnosis • Clinical management	• None • Present but with scarce supplies • No ECG for all MPCF. Patients were send to far away hospitals	• Complete free of charge treatment of ChD • Present with enough supplies • ECG for all MPCF by tele medicine
Financial Accessibility			
Direct costs and prices of services Indirect costs	• User fees • Source of financing of the MPCF • Opportunity costs of time • Transportation costs • Food and lodging	• None • Mainly Municipal. Insufficient to fulfill all the needs • Initially high • Initially present • Not considered	• None • Mainly Municipal. Some supplies financed by MS • Reduced • Excluded • Not considered
Acceptability			
Characteristics of health services	• In line with prevailing cultural norms	• Not explored	• Pilot tests to investigate this item. Need of specialized teams to explore this aspect more deeply
User’s attitudes and expectations	• Satisfaction with the health service	• Not explored	• Pleased to receive complete diagnosis and treatment free of charge in the first level • More studies needed to assure this item

Many aspects were evaluated and many of them showed the necessity to be scaled-up. The characteristics were ranked according to needs for improvement and strategic relevance for implementation of the ChD program. Specifically, the lack of good roads and adequate public transportation were identified as main access barriers since the traditional type of provision of health services in Argentina requires the movement of patients from one provider to another in order to complete the diagnostic and/or therapeutic process. Structural changes to solve this situation were not within the scope of the project but it was possible to change the way services were provided in order to be able to increase adhesion. Another important barrier identified was the lack of a healthcare network; therefore, it was necessary to work with the hospitals in order to receive those patients that required complex diagnostic procedures or specialized treatments that could not be provided in the first care level. Both of these were a priority because, if not, the ChD program could not have been implemented.

For the items selected as strategically relevant to the program, a set of possible improvements were designed and discussed with local authorities. Once an agreement on how to proceed was reached, MS helped implement the solution, if necessary with financial resources. The financial resources eventually provided were scarce (< USD$7000) in order to assure the sustainability of the project once MS was no longer involved. For structural problems out of the scope of the program, reports were generated and recommendations were made to local authorities.

With all the information gathered during this investigation, a “patient-centered” model was designed. All the services needed for basic diagnosis and treatment were done in the MPCFs. As a result of the implementation of the extended program, 17,894 patients were serological evaluated for ChD diagnosis, 1,394 were positive and were 1,035 treated with antitrypanosomal drugs. The specific survey to detect risk factors for ChD was performed in 3,750 children from 46 public schools, a total of 280 children were tested at the MPCFs.

The financing source of the MPCFs was mainly from the municipal budget (although some drugs and vaccines came from the National Ministry of Health). MS bought two electrocardiograms with telemedicine and paid extra salaries to four local physicians to coordinate specific actions: gather information about the number of people diagnosed and treated as well as basic data through the monitoring process. Initially, a cardiologist was hired by MS to read and analyze the electrocardiograms.

In Argentina, there are very few experiences in public – private associations and in implementation analysis. Given the lack of a referential framework, an extensive evaluation of implementation matrices was done (Table 3).

**Table 3:** Implementation related to the Intervention characteristics and outer setting

Topic	Baseline and basic aspects
I. Intervention Characteristics	
A. Intervention Source	Seen as externally developed by key stakeholders
B. Evidence Strength & quality	Few evidence supporting the belief that the intervention will have desired outcomes
C. Relative advantage	Stakeholders’ perception of the advantage of implementing was not clear at the beginning, although no alternative solution was available
D. Adaptability	Disposition to adapt and tailor the intervention to meet local needs
E. Trialability	A pilot project was approved to be done by local authorities
F. Complexity	Perceived as high by both key stakeholders
G. Design Quality and Packaging	The project was easy to understand and accessible to users
H. Cost	Although drugs were free of charge for the local authorities and patients, the needs to have other supplies increased costs.
II. Outer Setting	
A. Patient needs & resources	Barriers were analyzed, although until the pilot project implementation some were unknown
B. Cosmopolitanism	No network with other external organizations
C. Peer pressure	No competitive pressure
D. External Policy & Incentives	National law and guidelines for medical treatment. No local programs or primary care guidelines for diagnosis and medical treatment
III. Inner Setting	
A. Structural characteristics	A governmental organization with a small and inflexible budget
B. Networks & Communications	No social networks, informal communication channels within the organization
C. Culture	Inflexible organizational models, no possibility of hiring human resources, low salaries, lack of incentives
D. Implementation climate	Although perceived as a necessary intervention, many stakeholders felt that the main objectives were very difficult to achieve. Goals were clearly communicated and an intensive training program was planned to be carried on during the first years, including “hand on” practice, medical forum, and clinical coaching
E. Readiness for Implementation	Leadership engagement, available resources, and access to knowledge and information were assured
IV. Individuals	
A. Knowledge & beliefs about the intervention	Scarce knowledge about public health and public policies
B. Self-efficacy	Scarce knowledge about anti parasitic drug administration
C. Individual Stage of change and identification with organization. Other personal attributes	Health teams perceived the municipality as a difficult organizational structure to be changed, more political focused than involved in health policies. Low salaries and lack of incentives plus bad infrastructure in the MPCF made a complex situation
V. Process	
A. Planning	A good and simple scheme for implementing the ChD program was performed
B. Engaging	Some health teams were initially engaged due to their previous experience. MS worked hard in coaching health teams and solving every problem or doubt. A 24 h communication line was available for the entire centers with a MS physician
C. Executing	The pilot project was central for latter scaling up
D. Reflecting & evaluating	Quantitative and qualitative feedback about the progress and quality of implementation was delivered

Through this assessment, an important finding was an initial perception of all the members interviewed that some changes in the way of providing health services were needed. Nonetheless, all agreed that those changes were extremely difficult to achieve and there was no competitive peer pressure to enforce them. Another important finding was the lack of important barriers for the implementation and development of the project.

## Discussion

People with low income typically have diminished access to health care providers. When health care is needed and it is not available, people’s health worsens contributing to a further reduction in income and higher health related out-of-pocket payments, both of which negatively impact poverty [20]. This is one of the main reasons why granting access to healthcare is fundamental to end this vicious cycle.

As mentioned above, Argentina has free of charge public health governmental suppliers distributed all across the country with uneven levels of capacity, complexity and quality. While access to healthcare in Argentina is free of charge in public facilities, quality and availability of equipment as well as qualified human resources may represent challenges to PHGMs [21]. This complex structure results in diverse sanitary situations across the country which forces the design and implementation of public health policy initiatives to be tailor made.

A so called “patient centered model” resulted in a practical, low cost and easy way to minimize or reduce the healthcare barriers found during the survey. Nearly all the patients that were serologically evaluated knew about the disease and most had a family member or acquaintance with ChD. This level of awareness displayed by the patient pool improved the ability to execute the program and the willingness of the people to be evaluated. Many of these potential patients had already made medical consultations regarding ChD, most of which had gone unanswered by the medical staff. The program achieved a high rate of adhesion to the treatment, with very few drop-outs. The high retention rate is attributable to the fact that patients were evaluated with a high frequency strategy and there were social workers in charge of home visits if necessary.

In 2014, MS’ venture with the La Plata City was the only project focused on granting ChD patients access to ambulatory care facilities with high quality standards: providing safe, effective, timely, efficient and equitable treatment, with a unique people-centered model.

The main conclusions that derive from the analysis of access barriers for this project are that there are two critical aspects to focus on during implementation:1) strengthening resources of the primary healthcare centers, and 2) creating a patient centered clinical management. Although in Argentina patients typically switch from one provider to another in order to complete the entire diagnostic and therapeutic process, providers that are usually distant from each other, this traditional pattern was considered highly inconvenient for this project. The pilot project evidenced that continuing with a model of rotation of health centers for diagnosis and treatment could entail that, at least, 30% of patients would never have an ECG, which is fundamental for ChD diagnosis and treatment. Previous to implementation of the program, many ECGs were not performed because since there were no electrocardiographs available at the MPCFs, patients needed to go to the PHGMs to have an ECG done. This implicated extra days of absence from the workplace and therefor many patients did not get an ECG.

In terms of the implementation aspects, trust building became a key issue. A senior physician from MS was fully dedicated to the project and available on the phone for medical queries from MPCFs staff 24 h during business days. Although help was not often needed, high level of availability offered by MS’s staff was well received by MPCFs staff and helped to consolidate confidence between both entities.

As a response to limited and inflexible municipal budgets, MS decided to donate two electrocardiographs to La Plata Municipality and to invest in human resource training. MS considered these two actions as key to succeed in the implementation startup. All the financial investments made by MS along the implementation, mostly in human resources, were small and could be easily absorbed by municipal budgets, if necessary, to assure the project sustainability. MS identified a series of structural problems during the data analysis period, but the necessary actions to solve these problems were initially defined as out of scope. For example, lack of investment in infrastructure, problems in the hiring of human resources, etc., that did not interfere in the implementation of the project.

Before the final project was designed, the main difficulties were associated to the limited existence of evidence supporting the desired outcomes of the project which did not give the stakeholders a clear vision of implementation advantages. There was an additional challenge: key stakeholders perceived the intervention source as externally developed and health teams perceived that the municipality was a difficult organization to be changed. Low salaries and lack of incentives plus bad infrastructure in the MPCFs were also key issues.

When the pilot project began, the advantages of this new intervention became visible and the first results of the pilot project encourage both authorities and health teams, to scale the project.

After the implementation phase was launched, positive results were self-evident and sufficient to guarantee the initiative’s sustainability. By the end of 2014, the project became a Municipal Program in line with others like vaccination campaigns.

In 2015, 2 years after the implementation, MS was ready to leave the project in the hands of the municipality. However, in light of upcoming presidential elections and potential change in the local authority, MS decided to remain actively involved in the project. The rotation of local authorities was seen as a challenge to implementation continuity, both from a political willingness perspective as well as availability of resources and proper understanding of the program scope.

Although during the first year of rotation of local authorities the number of treated patients fell, the program was not interrupted and during the second year, the program showed an increase in treatment rates.

By the end of 2017, after the new administration took office and the program continuity was guaranteed, MS began to decline its participation in the project. Although MS’s participation is not necessary to assure the program continuity, local authorities invited MS to keep participating in the ChD program. Nowadays, MS’s activity is to supervise the general management of the program: new patients enrolled in the program, number of diagnostic procedure, number of treatments performed and act as an external consultant for complex clinical cases.

Retrospectively, the ChD program of La Plata was very successful since 17,894 people were tested, 1,394 were diagnosed and medically evaluated, 1,035 were treated for ChD and 3,750 children from public schools were evaluated for risk factors. Pillars of success were the deep knowledge of access barriers, a comprehensive approach considering strengths and weaknesses of the local healthcare system, and being able to design a reasonably tailored made project. Trust building among stakeholders and the implementing task force was another key success factor. Finally, the limitations of this study are the absence of specific studies to explore user’s attitudes and expectations, such as satisfaction with the health service, and how the program was in line with the prevailing cultural norms of the community.

## Conclusions

This is the first study that shows the feasibility of the primary care level to give high quality early diagnosis and timely treatment to a population with ChD in Argentina. Although further studies should be done to gather more evidence, the results from this study can encourage policy makers to give medical assistance to people in early stages of ChD without other severe illnesses in the first health care level. This can help to reduce the burden of this disease and to reduce congenital transmission.

Tailor made programs and public-private associations should be considered for vulnerable populations in emerging economies in order to enhance efforts and obtain better results. Public-private associations may constitute a challenge in low and middle –income countries due to the limited experience in health care systems with this type of associations, the lack of regulations and a complex government structure. Although these types of associations may have been applied in healthcare settings before, references on these experiences are not easily available in the literature. This program may be replicated in other countries of Latin America were Chagas is a main public health issue and, with the corresponding adaptations, for other neglected diseases as well. Some necessary conditions for successful implementation lie in the comprehensive analysis of access barriers and the implementation context. The results of this comprehensive analysis would be crucial for the development of a suitable project based on the local needs and the acceptability from the health teams and local authorities involved.

## Additional file

Additional file 1: Short survey used for children attending public schools. (PDF 192 kb)

## Data Availability

The datasets used and analyzed during the current study are not publicly available since they are from a governmental source but are available from the corresponding author on reasonable request.

## Abbreviations

ChD: Chagas disease
MLP: Municipality of La Plata
MPCF: Municipal Primary Care Facilities
NTD: Neglected Tropical Diseases
PHGM: Public Hospitals with Governmental Management
