# Supplementary material for: Building an innovative Chagas disease program for primary care units, in an urban non- endemic city
Source: BMC Public Health. 2019 Jul 8;19:904. doi: 10.1186/s12889-019-7248-5 (PMC6615298; doi:10.1186/s12889-019-7248-5)
Supplement: Supplementary file 1 — Short survey used for children attending public schools. (PDF 192 kb) [file 12889_2019_7248_MOESM1_ESM.pdf]

Code Number: School Number:

Grade: Turn: Date:

Questions for the pupil

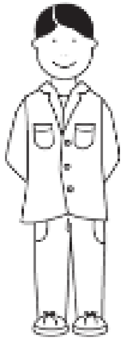

Name and Surname:

DNI: Date of birth:

Phone number:

Place of birth: Province of Argentina

- ○ Bolivia ○ Paraguay ○ Peru ○ Other country

Had you ever receive a blood transfusion? ○ No ○ Yes

Questions for the mother

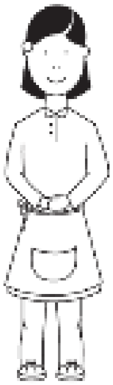

Name and Surname:

DNI: Date of birth:

Phone number:

Place of birth: Province of Argentina

○ Bolivia ○ Paraguay ○ Peru ○ Other country

Had you ever receive a blood transfusion? ○ No ○ Yes

Have you had a positive blood test for Chagas disease during your pregnancy?

Yes ○ No ○
